# Assessing canalisation of intraspecific variation on a macroevolutionary scale: the case of crinoid arms through the Phanerozoic

**DOI:** 10.7717/peerj.4899

**Published:** 2018-05-31

**Authors:** Catalina Pimiento, Kit Lam Tang, Samuel Zamora, Christian Klug, Marcelo R. Sánchez-Villagra

**Affiliations:** 1 Museum für Naturkunde, Leibniz Institute for Evolution and Biodiversity Science, Berlin, Germany; 2 Smithsonian Tropical Research Institute, Balboa, Panama; 3 Paleontological Institute and Museum, University of Zurich, Zurich, Switzerland; 4 Instituto Geológico y Minero de España, Zaragoza, Spain

**Keywords:** Disparity, Variability, Macroevolution, Phanerozoic

## Abstract

Clades that represent a new ‘Bauplan’ have been hypothesised to exhibit more variability than more derived clades. Accordingly, there is an expectation of greater variation around the time of the origin of a clade than later in its evolutionary history. This ‘canalisation’ has been tested in terms of morphological disparity (interspecific variation), whereas intraspecific variation in macroevolution is rarely studied. We analysed extensive data of brachial counts in crinoid populations from the Ordovician to the Recent to test for canalisation in morphological intraspecific variation. Our results show no support for the canalisation hypothesis through the Phanerozoic. This lack of pattern is maintained even when considering crinoid subclades separately. Our study is an example of the lack of universality in such macroevolutionary patterns both in terms of organisms and in terms of modules within them. It is also an example on the challenges and limitations of palaeontological studies of macroevolutionary processes.

## Introduction

It has been proposed that the extent of variation within a species is determined by generative processes that are tied to the history of the clade (Erwin, 2007): clades that represent a new ‘Bauplan’ have more variability (i.e., more capacity to generate variation), whereas more derived clades have been canalised in such a way that they are less prone to vary (Waddington, 1957). Canalisation implies robustness of the phenotype. Flatt (2005: 288) defined it as “the reduced sensitivity of a phenotype to changes or perturbations in the underlying genetic and nongenetic factors (e.g., the environment) that determine its expression”. This assumption is at the centre of a major and contested hypothesis on the pattern of evolution in geological time: morphological variability among species appears before taxonomic diversity (Erwin, 2007).

The amount of change in morphospace occupation across species—interspecific variation or disparity—has been examined in both palaeontology (Foote, 1996; Hughes, Gerber & Wills, 2013; Benton, Forth & Langer, 2014; Lloyd, 2016) and neontology (Harmon et al., 2010). In contrast, morphological intraspecific variation in macroevolutionary timescales, and with that canalisation, remains largely unexamined. Palaeontological studies that have addressed intraspecific variation have been carried out mainly on microevolutionary scales (e.g., Frey, Maxwell & Sánchez-Villagra, 2016) or have examined large clades in part of their evolutionary history (De Baets, Klug & Monnet, 2012; De Baets et al., 2015). To our knowledge, only one study (i.e., Webster, 2007) has examined intraspecific variation on a macroevolutionary scale by studying a whole major clade—a ‘Bauplan’—along its entire range. That is the case of trilobites, a group restricted to the Palaeozoic. In agreement with the canalisation hypothesis, Webster (2007) reported more polymorphism in early and middle Cambrian trilobites than in any subsequent periods of their evolution; a pattern that coincides with the large overall disparity of the group over time (Hughes, 2007; Hunt, 2007).

Crinoids can serve as a case study clade to assess canalisation in intraspecific variation across geological time scales because they are a monophyletic group that preserves well in the fossil record (sometimes in reasonably large groups entombed by obrution events and likely representing the same or coeval populations) and because unlike trilobites, they span most of the Phanerozoic persisting until the Recent (e.g., Hess et al., 1999). Here, based on an extensive global museum survey, we compiled a comprehensive dataset of populations of fossil and modern crinoids spanning from the Ordovician to the Recent. We used crinoid arms, specifically brachial counts, to test the hypothesis of canalisation in intraspecific variation in a macroevolutionary timescale. Our results contrast with the trilobite case and serve to illustrate the challenge of generating universal principles in macroevolution, in particular for aspects in which ecology, development and chance play a role (Benton, 2009). Likewise, our study serves to point out the methodological challenges involved in the macroevolutionary studies of intraspecific variation.

## Materials and Methods

We gathered data through extensive work in museum collections, with additional data obtained from online, digitized collections and scientific literature. Our search was made based on specimen availability, and not based on finding a broad range of taxa. Therefore, we considered our search efforts to be random to some extent, although we tried to cover the studied time interval well with samples covering the Ordovician to Recent. In all cases, we examined the specimens and selected well-preserved populations, i.e., groups of specimens of the same species at a single site, with calyx and arms preserved. We collected data from 15 collections in seven countries: University of Zurich’s Paleontological (PIMUZ) and Zoological (ZMUZ) Museums; Natural History Museum of Basel (NMB); Institut für Geowissenschaften, Tübingen (GPIT); Senckenberg Natural History Museum Frankfurt (SMF); Muschelkalkmuseum Ingelfingen (MHI); Stuttgart State Museum of Natural History (SMNS); The British Natural History Museum (NHM); Museum für Naturkunde, Berlin (PMB); Le Muséum National d’Histoire Naturelle, Paris (MNHN); Swedish Museum of Natural History (NMG); Zoological Institute of the Russian Academy of Sciences (ZIN); Natural History Museum of Geneva (MHNG); Oxford University Museum of Natural History (OUMNH); and Smithsonian Institution National Museum of Natural History (USNM). We took photographs of all selected specimens (supplemental material). Further, we added data on additional specimens in iDigBio (http://www.idigbio.org/) using the following criteria: Scientific Name: Crinoidea, filtered by “must have media”. All images gathered from iDigBio come from the Yale Peabody Museum (YPM) and the USNM. Further, we searched online for publications containing images of additional populations of specimens. Additional specimens examined are listed in the supplementary information.

**Figure 1:**
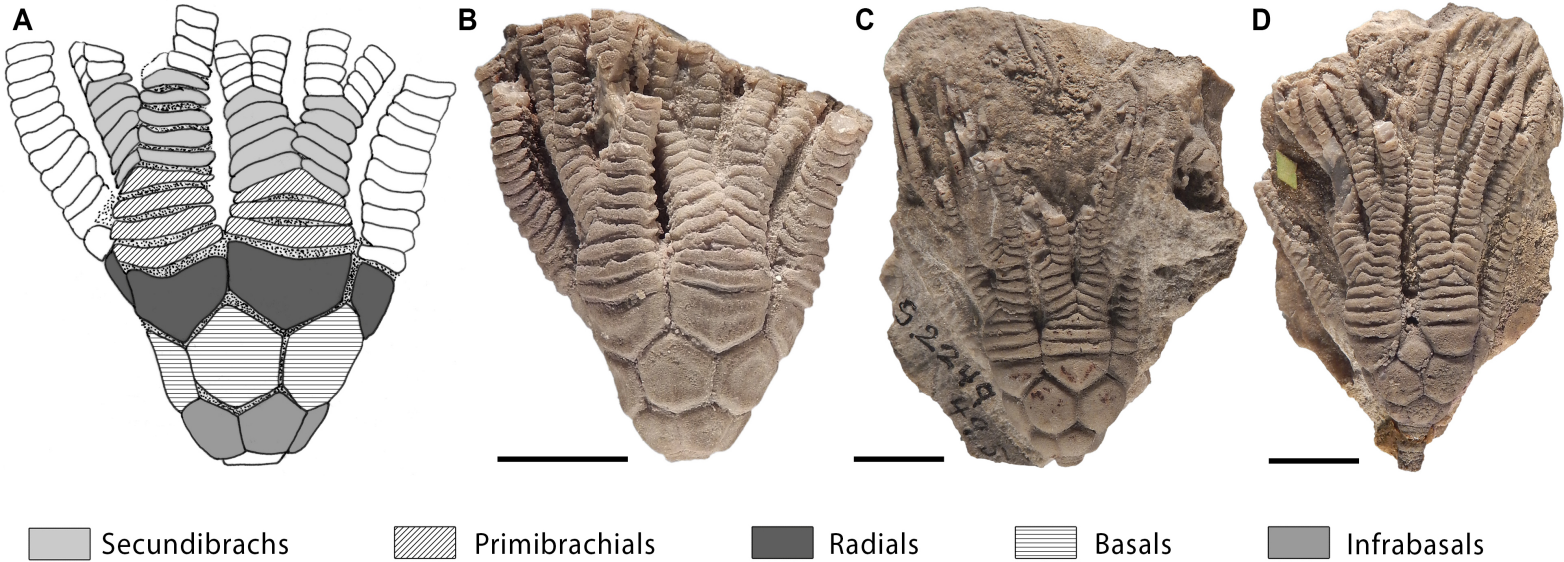
Intraspecific variation in primibrachials of *Cupulocrinus jewetti*. Crinoid *Cupulocrinus jewetti* (USNM S2249) from the Ordovician of Kentucky (USA) showing intraspecific variation in the number of primibrachials. (A) Camera lucida drawing of specimen figured in (B) with indication of anatomical parts (only two frontal arms coloured). (B–D) Different specimens of *C. jewetti* showing variation in the number of primibrachials, ranging from three to five per branch. All scale bars represent 1 cm. Photographer: S Zamora.

For each studied specimen, we counted the number of primibrachials and secundibrachials per arm; however, we only used primibrachials (either fixed or free brachials) in our analyses (Fig. 1). Primibrachials are brachial structures that start from the radials and that include the first axillary plate (Ubaghs, 1978). Brachial numbers are a good proxy for intraspecific variation because arms inserted in radials and radial plates are generally thought to be homologous across crinoid clades (Guensburg & Sprinkle, 2003; but see Simms, 1993 for an alternative scheme). Primibrachials are usually easy to recognize and their number can be quantified from the earliest crinoids to modern ones. They also form part of the proximal portion of the arm that generates early in ontogeny, and it is less prone to predation/regeneration than distal parts (Baumiller & Gahn, 2004). All these homologisation, taphonomical and pragmatic considerations make primibranchial numbers an excellent subject of study for this large-scale kind of study requiring sampling of populations. Future studies could address another module of crinoids, namely the calyx, with the advantage that this complex structure is less prone to predation/regeneration than the proximal arms. However, homologisation and recording of numbers in primibrachials is less challenging than the study of calyx plates (cf. Guensburg & Sprinkle, 2003; Simms, 1993; Wright, 2015).

The data from museum collections were taken on site, whereas the rest were gathered from images. For each specimen, we examined the labels and used collection databases to verify their age. In total, we gathered counts from 1,283 specimens of 251 species that range from the Ordovician to the Recent (485–0 Ma; Gradstein et al., 2012; Table S1; complete dataset available from the Dryad Digital Repository http://datadryad.org/review?doi=doi:10.5061/dryad.vh0qt).

**Figure 2:**
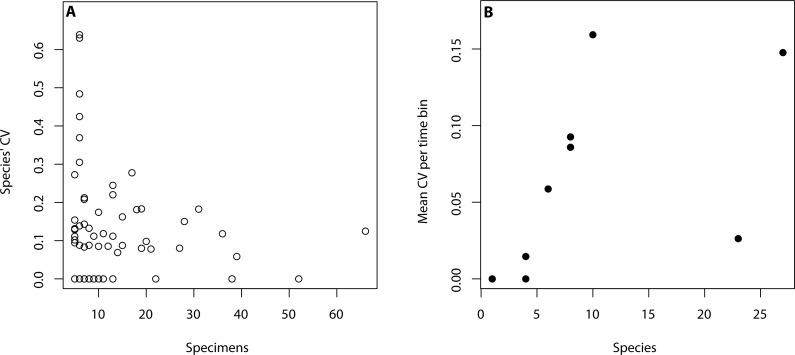
Relationship between intraspecific variation and sampling size. (A) Intraspecific variation is measured as species’ coefficient of variation (CV) and sample size is regarded as the number of specimens per species (adjusted R2 = −0.01, *p*-value: 0.9). (B) Intraspecific variation is measured as mean CV per time bin and sample size is regarded as the number of species per time bin (adjusted R2 = 0.12, *p*-value: 0.18).

All analyses were performed in R (R Core Team, 2015) using only primibrachial counts as they were countable throughout our sample. The R code is available in the supplemental material. To assess intraspecific variation, we considered species where five or more individuals were available aiming to capture variation within populations. This subset of data consists of 91 species and 1,018 specimens (Table 1). Based on this dataset, we created a data frame with the primibrachial counts per arm of each species. We then calculated the mean, range (max-min), standard deviation and coefficient of variation (herein, CV) and number of individuals per species (Table S1). To calculate the mean of each species we summed the primibrachial counts of all arms of all individuals and divided it by the number of arms. Time was binned as follows: Ordovician, Silurian, Devonian, Carboniferous, Permian, Triassic, Jurassic, Cretaceous and Cainozoic. For our analyses, we used the mean age of each bin following Gradstein et al., 2012. To assess for the effect of sample size differences across species and time periods (Table 1), we tested for a linear correlation between species’ CV and the number of individuals sampled per time bin (Fig. 2A), and between mean CV values and number of species per time bin (Fig. 2B). We found that intraspecific variation and sample size do not correlate in either case (see caption in Fig. 2). However, it became apparent that a lower number of specimens yields higher intraspecific variation.

**Table 1:** Number of species and specimens per geological period. Only those species with more than five individuals were considered for the intraspecific variation analyses.

Period	Total no. species	Total no. specim.	No. species ≥5 specim.	≥5 specim.
Ordovician	19	149	10	126
Silurian	23	124	8	96
Devonian	14	103	8	91
Carboniferous	102	322	27	205
Permian	42	74	4	27
Triassic	9	91	4	81
Jurassic	7	159	6	147
Cretaceous	2	14	1	11
Cainozoic	29	249	23	234
**Total**	251	1,283	91	1,018

We plotted the CV values versus time, and applied both a linear regression to statistically test for a decline in variation over time, and a local fitting regression (LOESS) to assess for a more general trend (Kohn, Schimek & Smith, 2000). We further applied the methods of Hunt (2006), Hunt (2008) and Hunt & Carrano (2010), which tests for models of trait evolution while accointing for sampling differences across the time series. Accordingly, we calculated the CV mean, variance, and number of species in each time bin. We then tested for the following common models of trait evolution: Random walk (UWR), where evolutionary increments are independent and equally likely to increase or decrease; directional evolution (GWR), which features a trend of increasing or decreasing trait values over time (here, the case of decreasing in trait (CV) value would be and indicator of canalisation); and stasis, with trajectories that show fluctuations around a steady mean. We used the R package *paleoTS* (Hunt, 2008) to fit these models to our time series. This package uses maximum-likelihood estimation to fit these models, and the small-sample size Akaike Information Criterion (AICc) as a measure of model support (Hunt & Carrano, 2010). The interpretation of these scores is aided by Akaike weights, which are the proportional support that each model receives.

In order to assess interspecific variation, we created a data set as described above, but used all specimens relaxing the ‘5 specimens’ criterion above, and calculated the range of primibrachial counts per arm for each species. As in Foote (1999), we regarded the mean pairwise character (range of primibrachial elements) distance between species over time as a proxy for disparity. In so doing, we binned our time series using the mid age of each geological Period assigned to each species. We created a species distance matrix using the functions “dist” in R and calculated the mean pairwise distance separating species in time bins using the “mpd” function of the R package picante. Finally, we tested for a correlation between intra- and interspecific variation (mean CV per time period vs. mean pairwise distance) over time using the generalized differences method (McKinney, 1990) as implemented in the R function “gen.diff” provided by Graeme Lloyd (http://www.graemetlloyd.com/methgd.html).

## Results

**Figure 3:**
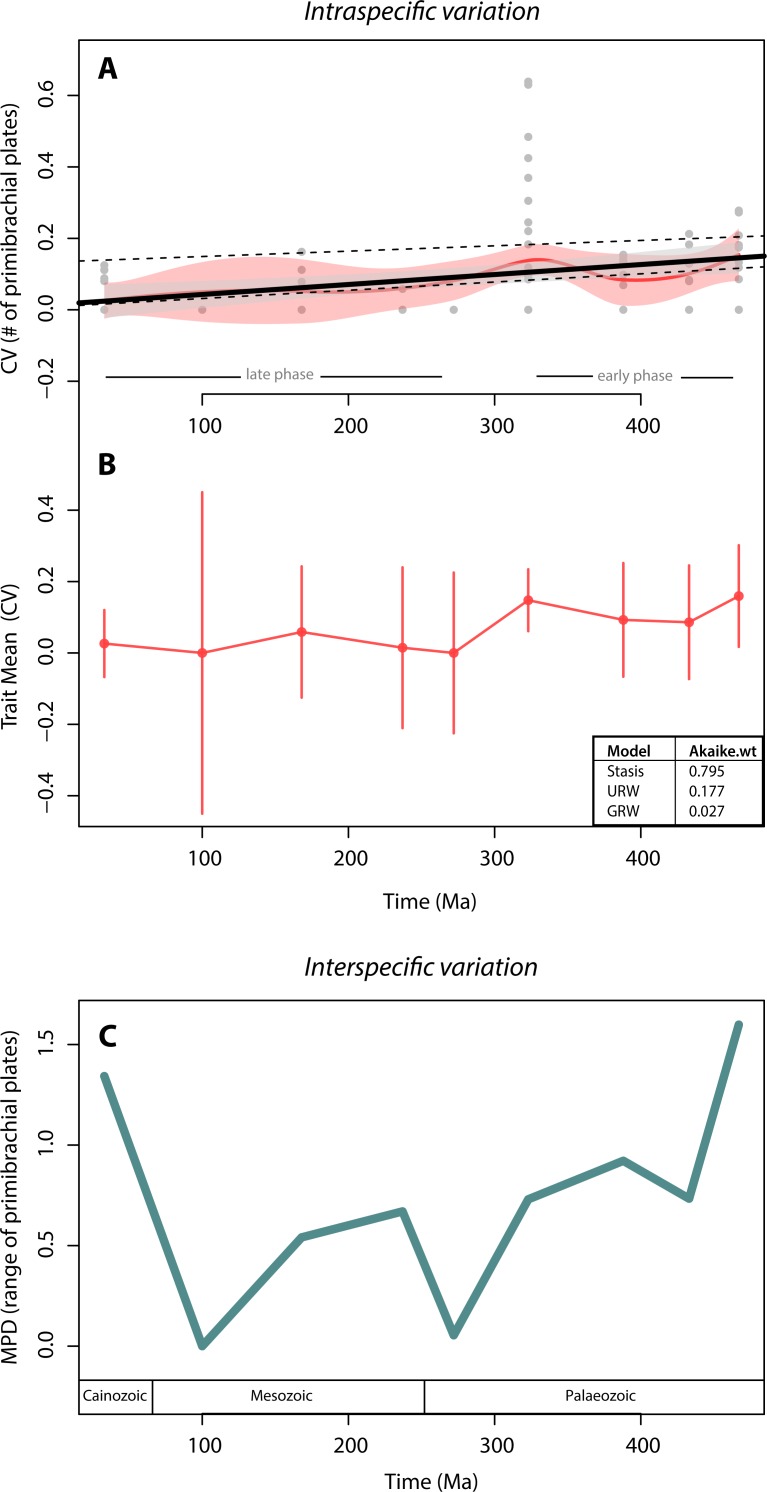
Crinoid intra- and inter-specific variation throughout the Phanerozoic. (A) Intraspecific variation measured as the coefficient of variation (CV = standard deviation/mean) through time bin. Black solid line shows the linear regression (R-squared = 0.10; *p*-value = 0.001). Grey polygon denotes the 95% confidence intervals of the linear regression. Upper dashed line shows the linear regression excluding invariant species (CV = 0). Lower dashed line shows the linear regression excluding the extremely high CV values of the Carboniferous (323 Ma). Red curve shows the local regression fitting (LOESS), and the red polygon shows its confidence intervals. (B) Intraspecific variation (CV) trajectory over time. Bars represent standard errors on the mean. Inserted table show support of three evolutionary models tested (C). Interspecific variation measured as the mean pairwise character (=range of the number of primibrachials) distance (MPD) between species over time. Time bins are shown as mid age (see Methods).

We found no support for canalisation in intraspecific variation on a geological time scale, using segmental structures of crinoids as subject of investigation. This is indicated by (1) the lack of a trend in variation (CV) through time, as evidenced by both the linear and the local regressions (Fig. 3A), and (2) the lack of support for the GWR evolutionary model as evidenced by its low AIC weight (Fig. 3B). Indeed, when testing for the three models of trait evolution, stasis was the model that best fit our data, accounting for 80% of the Akaike weight and greatly out-performing the UWR and GWR models. Further, although there is a statistically significant correlation between intraspecific variation (CV) and time (thick black line in Fig. 3A; adjusted R squared = 0.10; *p*-value = 0.001) our extensive dataset poorly explains this trend. The noisiness and high variability of the data is evidenced by the fact that most data points lay outside the confidence interval of the linear regression (grey polygon in Fig. 3A). The significant predictive power of the linear model (as evidenced by the *p*-value) and the poor fitting of the data in the model (as evidenced by the *R*^2^ value) is a counterintuitive result that may be due to two issues: (1) 70% of species sampled have zero variation (i.e., most crinoid species have invariant primibrachials), and (2) there is a particular time bin (the Carboniferous) that is characterized by extremely high intraspecific variation.

To assess for the potential effects of these two data features, we applied a linear regression to two subsets of data: one excluding the non-variant species (CV = 0) and one excluding the extremely high values from the Carboniferous. Excluding the non-variant data results in a much poorer model fit and a non-significant correlation (upper dashed line in Fig. 3A; adjusted R squared = 0.005; *p*-value = 0.32), and therefore, the lack of variation of most species does not make the linear regression worse (if anything it improves it). Further, the removal of extremely high CV values produces similar results than when using the full set of data, except that there is a slightly better model fit (lower dashed line in Fig. 3A; adjusted R squared = 0.27; *p*-value <0.001), and therefore, the extreme variation of the Carboniferous pulls the data into a bad linear model fitting, although not dramatically (from 10% to 27%).

The local regression (red curve in Fig. 3A) further reinforces the lack of a clear pattern, as it is predominately flat in two distinct phases: in the early phase, from the Ordovician to the Carboniferous (485–323 Ma), there is a slightly higher intraspecific variation than during the rest of the time series. The highest variations (CV = 0.6 by *Eratocrinus salemensis* and *Sarocrinus nitidus*), however, do not occur temporally close to the origin of the clade. Instead, they fall during the Carboniferous (∼323 Ma). This peak in variation is followed by a reduction that extends to the Permian (∼272 Ma). The late phase, from the Permian to the Cainozoic, is characterized by low and relatively steady intraspecific variation. As shown in Fig. 2, the highest level of variation is not determined by sample size. The two species that vary the most are both sessile Cyathoformes, which live in high energy siliciclastic environments with subsidiary carbonate facies (Kammer, Baumiller & Ausich, 1998).

Additional per clade analyses showed that the lack of a clear pattern of canalisation is not evident in any independent phylogenetic group of crinoids (Table 2). Linear regressions are notably variant, with Pentacrinoidea being the only group that presents a statistically significant correlation (*p* < 0.05). Adjusted *R*^2^ values are remarkably low, ranging from −0.03 (Articulata and Cyathoformes) to 0.18 (Monobathrida). Further, there was no support for the the GWR model in any independent group, with stasis and URW being the models that best fit our per-group data (Table 2).

**Table 2:** Per clade linear regression and tests of evolutionary mode. Diplobathrida and Disparida subclades did not have enough data points to perform any analyses. Dash indicates lack of enough data for model testing.

	Group	*R*^2^	*p*-value	Best model fit	Akaike.wt
‘Subclass’	Pentacrinoidea	0.140	0.0003	Stasis	0.795
	Camerata	0.108	0.171	–	–
‘Subclade’	Ariculata	−0.026	0.689	URW	1
	Cyathoformes	−0.034	0.853	URW	0.973
	Flexibilia	−0.071	0.661	–	–
	Monobathrida	0.179	0.122	–	–

In terms of disparity, we found a general decrease in interspecific variation throughout the Palaeozoic and strong fluctuations afterwards. The highest interspecific variation values take place at the beginning (Ordovician) and the end (Cainozoic) of the clade. However, similar to the pattern for intraspecific variation, there is no clear trend (Fig. 3C). We further found a non-significant correlation between intra- and interspecific variation (rho = 0.57; *p*-value = 0.15).

## Discussion

Our results suggest that there was no canalisation in crinoid intraspecific variation. Instead, stasis was the general evolutionary mode (i.e., no net changes over time, Fig. 3B). Most of the mean variation values fall around zero, indicating that in general, low instraspecific variation was maintained over time. The lack of canalisation interpreted from the evolutionary mode (which accounts for the mean variation, its volatility and the sample size Hunt & Carrano, 2010; Fig. 2B) is corroborated by the examination of all variation points across the time series (Fig. 2A). Accordingly, the extensive set of data here studied (1,017 specimens of 91 crinoid species encompassing most phylogenetic groups across the entire Phanerozoic) only explained 10% of the (significant) correlation between intraspecific variation and time (Fig. 3A). This bad model fitting is not driven by the lack of intraspecific variation of most species, and it is somewhat improved (from 10% to 27%) when excluding highly variant species. We interpret this counterintuitive (i.e., significant correlation and bad model fitting) result as a lack of clear pattern of canalisation in intraspecific variation, in contrast to what has been hypothesised as a universal pattern to be expected in the evolution of ‘Bauplans’. Alternatively, we found that there are two general phases in which there is higher intraspecific variation in early evolutionary stages, and lower variation in late evolutionary phases. This interpretation; however, does not correspond to canalisation, as the highest variation displayed in primibrachial plates does not occur at the beginning of the clade (Ordovician), but ∼162 mys later (Carboniferous). Our results and interpretations are supported by per-group analyses showing that the lack of a pattern is consistent across crinoid groups (Table 2), further evidencing that the potential for canalisation is not being masked by independent patterns in phylogenetic groups.

Our results contrast with the trilobite pattern (Webster, 2007); however, there are differences between the two studies. First, Webster’s work was based on polymorphism in cladistic datasets, whereas ours was on the coefficient of variation of one anatomical region (i.e., primibrachials). Second, the stratigraphic range of the organisms studied differ: trilobites originated in the Cambrian—a period known for higher disparity and developmental experimentation (Gould, 1989)—and went extinct in the Permian (Owens, 2003), whereas crinoids originated in the early Ordovician and persisted until the Recent (Guensburg & Sprinkle, 2001).

Canalisation in trilobites, i.e., higher intraspecific variation at the beginning of the clade, coincides with their high disparity during the Cambrian (Hughes, 2007; Hunt, 2007). Crinoids also display their highest disparity at the beginning of their evolutionary history (Ordovician, Foote, 1994; Peters & Ausich, 2008; Deline & Ausich, 2011); nonetheless, this is decoupled from the intraspecific variation, which peaks in the Carboniferous, ∼162 Ma after their origination. The claims on high disparity being associated with high intraspecific variation have been made in relation to the Cambrian and the origin of new ‘Baupläne’ at the very beginning of the Phanerozoic (Erwin & Valentine, 2013). Because crinoids originated during the Ordovician, one consideration is that despite extensive time range studied here, our data do not capture the variation of the Cambrian, which is a critical time period in animal evolution, and therefore, that canalisation hypothesis does not apply to groups that evolved thereafter. Indeed, it has been proposed that Ordovician crinoids display higher variation in calyx-plating, plate arrangement in the stalk and possibly also the arms (Guensburg & Sprinkle, 2001; but see Guensburg, 2012; Wright, 2017). Hence, it is possible that that canalisation was already ‘completed’ when the first crinoids appeared in a strict systematic sense.

Another potential explanation for the lack of a decrease in intraspecific variation in crinoids during the Phanerozoic could be related to ecological factors. Crinoids display their highest diversity at the time when intraspecific variation peaks, during the Carboniferous, particularly in the Mississippian, (the so-called “age of the crinoids”, Kammer & Ausich, 2006). It has been suggested that higher intraspecific variation takes place at the beginning of clade because there are more “empty niches”, and as a result, more potential for diversification (Erwin, 2007). The diversification of crinoids in the Mississippian has been proposed to be the result of predatory release, i.e., the extinction of their vertebrate consumers (Sallan et al., 2011). Accordingly, the hypothesised lack of canalisation reported here can be due to the fact that the ecological opportunities enabling crinoid diversification did not take place early in their evolutionary history, but ∼162 mys later (Ausich & Kammer, 2013). Consequently, that stochastic favourable ecological conditions, and ultimately diversification potential rather than morphological disparity (Fig. 3B), drive intraspecific variation in crinoids.

Our data show that crinoids do not display higher intraspecific variation at the beginning of the evolutionary history, nor they display a decrease in variation over time. We interpret this result as lack of canalisation in morphological variation, and offer two potential explanations: (1) that canalisation was already completed when crinoids originated in the Ordovician; and (2) that intraspecific variation occurs when ecological conditions enable diversification, which can take place stochastically, at different times of an animal’s evolutionary history. However, because there are other (intrinsic and extrinsic) factors that could affect, and even bias the pattern (or lack thereof) observed, our interpretations warrant some caution.

Extrinsic factors (e.g., those not inherent to species traits) such as food availability, water temperature and energy, diseases and injuries, as well as taphonomy, sampling, and taxonomic misidentifications can affect within-species morphological variation. Accordingly, it is difficult to explain the lack canalisation without being able to disentangle the potential extrinsic drivers of variation. Another consideration is the relatively low diversity and abundance that characterizes the beginning of any clade, and its effects on sampling probabilities and therefore, on the detection of variation. More empirical studies are needed to be able to assess the universality of such patterns; however, future works should consider the complexity of the mechanisms at ecological, developmental and evolutionary scales, while taking into account the effect of taxonomy and taphonomy.

Intrinsic factors (e.g., those related with species’ morphological traits) can also play an important role. For instance, because the number of primibrachials is frequently used as a diagnostic character at high taxonomic levels (e.g., genera and families, see Moore & Teichert, 1978; Cole, 2017) the lack of clear pattern of canalisation in intraspecific variation in crinoids could be a taxonomic artefact. Another consideration is that different clades (and subclades) fixed for the number of primibrachials wax and wane through time and that the pattern that arises when counting primibrachials can be unrelated to the evolution of body plans. Although a large part of our data presents no variation, we did find that some clades exhibit more variation in brachial counts than others. Likewise, the characteristic number of segments in crinoid arms, its fixation in some clades and changes among them is indeed a pattern across large evolutionary time and one that deserves explanation. Future studies should examine different traits in different clades, such as the number of interradial plates in camerates, number of posterior plates among cladids (Wright, 2015), number of secundibrachials (or higher brachitaxes), or even total number of calyx plates (Simpson, 2010), as the degree of intraspecific variation is likely be clade-dependent.

Finally, because of modularity, the use of a single trait (e.g., Harmon et al., 2010) in this case, brachial counts, to investigate a general macroevolutionary pattern could impose important biases (as discussed by Webster (2015) in his study of intraspecific variation in Cambrian trilobites) because it could imply seeking for a local rather than universal pattern (Deline & Ausich, 2017; Hopkins, 2017). We consider that the study of a single trait presents advantages when it is based on an extensive temporal and taxonomic examination of heritable structures that can vary in traceable ways. Further, the examination of brachial counts has many advantages for the investigation of macroevolutionary patterns. First, they are analogue to other segmental structures that have been studied in macroevolution, i.e., the axial skeleton of vertebrates (Müller et al., 2010) and the segments of trilobites (McNamara, 1986; Jablonski & Bottjer, 1990; Hughes & Chapman, 1995). Second, segmental structures can be easily homologised and are a straight-forward subject to quantify variation. Nevertheless, it should be noted that such a system is no substitute for a more holistic examination of the organisms and represents thus a limited aspect of the whole variation.

The idea of canalisation, i.e., decrease in morphological variation in the evolution of a major clade (Webster, 2007), deserves scrutiny as it concerns a fundamental aspect of macroevolution. Canalisation has been hypothesised to evolve as a result of growing insensitivity of a genetic-developmental network, i.e., transcriptional regulators (Siegal & Bergman, 2002), that become more complex with time. Progress in understanding these principles has been made mostly on systems involving metabolic pathways (Wagner, 2005). The time is ripe to expand this to analyses of organismal systems (Rasskin-Gutman & Esteve-Altava, 2014). How the evolution of intraspecific variation is related to the overall disparity of the clade and has been remained largely unexplored. Our study of an important morphological feature in the evolution of crinoids through most of the Phanerozoic is a step in this direction.

## Conclusion

Crinoids display neither higher intraspecific variation at the beginning of their evolutionary history nor a decrease in variation over time (Fig. 2). We therefore reject the hypothesis of canalisation in morphological variation for primibrachial counts in crinoid populations through the Phanerozoic. Our results (based on a comprehensive dataset, which is available online and includes photographs of all specimens) are supported by per-group analyses showing consistency across crinoid groups (Table 2). This study contrasts with the single previous work that tested this idea on a macroevolutionary scale in trilobites (Webster, 2007), offering an example of the lack of universality in macroevolutionary patterns.

##  Supplemental Information

10.7717/peerj.4899/supp-1 Supplemental Information 1 R code R code—intraspecific variation of primibrachials in the Phanerozoic.

10.7717/peerj.4899/supp-2 Supplemental Information 2 All data collected and used in analysis using the R script included

10.7717/peerj.4899/supp-3 Table S1 Summary of all species sampled, their age, taxonomic affiliations and the proxies of variation Grey font denote species with less than 5 individuals, which were excluded from the intraspecific variation analyses.

## Additional Information and Declarations

### Data Availability

The following information was supplied regarding data availability: Dryad DOI: 10.5061/dryad.vh0qt.
