# Supplementary material for: Assessing canalisation of intraspecific variation on a macroevolutionary scale: the case of crinoid arms through the Phanerozoic
Source: PeerJ. 2018 May 31;6:e4899. doi: 10.7717/peerj.4899 (PMC5985148; doi:10.7717/peerj.4899)
Supplement: Table S1 — Grey font denote species with less than 5 individuals, which were excluded from the intraspecific variation analyses. [file peerj-06-4899-s003.docx]

**Supplementary Information**

**Assessing canalisation of intraspecific variation on a macroevolutionary scale: The case of crinoid arms through the Phanerozoic**

Catalina Pimiento^1,2^ , Kit Lam Tang^3^, Samuel Zamora^4^, Christian Klug^3^, Marcelo R. Sánchez-Villagra^3^.

**Table S1.** Summary of all species sampled, their age, taxonomic affiliations and the proxies of variation. Grey font denote species with

less than 5 individuals, which were excluded from the intraspecific variation analyses. sd = standard deviation; cv = coefficient of variation.

| **Species** | **Period** | **Epoch** | **Subclass** | **Clade** | **Primibrachial count** | | |  | **N** |
| --- | --- | --- | --- | --- | --- | --- | --- | --- | --- |
|  |  |  |  |  | **range** | **mean** | **sd** | **CV** |  |
| *Metacrinus_rotundus* | Recent | Recent | Pentacrinoidea | Articulata | 5 | 6.70 | 0.84 | 0.12 | 66 |
| *Pentacrinites_dargniesi* | Jurassic | Bajocian | Pentacrinoidea | Articulata | 0 | 2.00 | 0.00 | 0.00 | 52 |
| *Traumatocrinus_hsui* | Triassic | Ladinian | Pentacrinoidea | Articulata | 1 | 2.01 | 0.12 | 0.06 | 39 |
| *Traumatocrinus_hsui* | Triassic | Late Triassic | Pentacrinoidea | Articulata | 0 | 2.00 | 0.00 | 0.00 | 39 |
| *Pentacrinites_fossilis* | Jurassic | Sinemurian | Pentacrinoidea | Articulata | 0 | 2.00 | 0.00 | 0.00 | 38 |
| *Iocrinus_subcrassus* | Ordovician | Katian | Pentacrinoidea | Disparida | 3 | 4.16 | 0.49 | 0.12 | 36 |
| *Temnocrinus_tuberculatus* | Silurian | Wenlock | Pentacrinoidea | Flexibilia | 2 | 2.14 | 0.39 | 0.18 | 31 |
| *Codiacrinus_schultzei* | Devonian | Emsian | Pentacrinoidea | Cyathoformes | 3 | 2.97 | 0.45 | 0.15 | 28 |
| *Saracrinus_nobilis* | Recent | Recent | Pentacrinoidea | Articulata | 3 | 4.02 | 0.32 | 0.08 | 27 |
| *Encrinus_liliiformis* | Triassic | Anisian | Pentacrinoidea | Articulata | 0 | 2.00 | 0.00 | 0.00 | 22 |
| *Encrinus_liliiformis* | Triassic | Middle Triassic | Pentacrinoidea | Articulata | 0 | 2.00 | 0.00 | 0.00 | 22 |
| *Chariocrinus_andreae* | Jurassic | Bajocian | Pentacrinoidea | Articulata | 1 | 2.03 | 0.16 | 0.08 | 21 |
| *Moroccocrinus_ebbighauseni* | Devonian | Famennian | Pentacrinoidea | Cyathoformes | 1 | 1.96 | 0.19 | 0.10 | 20 |
| *Sagenocrinites_expansus* | Silurian | Wenlock | Pentacrinoidea | Flexibilia | 1 | 1.98 | 0.16 | 0.08 | 19 |
| *Pachylocrinus_aequalis* | Carboniferous | Mississippian | Pentacrinoidea | Cyathoformes | 1 | 2.09 | 0.30 | 0.14 | 19 |
| *Pachylocrinus_aequalis* | Carboniferous | Tournaisian | Pentacrinoidea | Cyathoformes | 2 | 1.92 | 0.37 | 0.19 | 19 |
| *Cupulocrinus_jewetti* | Ordovician | Darriwilian | Pentacrinoidea | Cyathoformes | 3 | 3.47 | 0.70 | 0.20 | 18 |
| *Cupulocrinus_jewetti* | Ordovician | Ordovician | Pentacrinoidea | Cyathoformes | 2 | 3.72 | 0.61 | 0.16 | 18 |
| *Cupulocrinus_angustatus* | Ordovician | Richmondian | Pentacrinoidea | Cyathoformes | 4 | 3.88 | 1.08 | 0.28 | 17 |
| *Taxocrinus_multibrachiatus* | Carboniferous | Serpukhovian | Pentacrinoidea | Flexibilia | 2 | 3.00 | 0.26 | 0.09 | 15 |
| *Pentacrinites_doreckae* | Jurassic | Sinemurian | Pentacrinoidea | Articulata | 2 | 2.00 | 0.32 | 0.16 | 15 |
| *Taxocrinus_stuertzi* | Devonian | Emsian | Pentacrinoidea | Flexibilia | 1 | 2.96 | 0.20 | 0.07 | 14 |
| *Scyphocrinites_sp* | Silurian | Pridoli | Camerata | Monobathrida | 0 | 2.00 | 0.00 | 0.00 | 13 |
| *Abrotocrinus_unicus* | Carboniferous | Mississippian | Pentacrinoidea | Cyathoformes | 1 | 1.06 | 0.25 | 0.23 | 13 |
| *Abrotocrinus_unicus* | Carboniferous | Serpukhovian | Pentacrinoidea | Cyathoformes | 0 | 1.00 | 0.00 | 0.00 | 13 |
| *Scytalocrinus_robustus* | Carboniferous | Mississippian | Pentacrinoidea | Cyathoformes | 1 | 1.07 | 0.26 | 0.24 | 13 |
| *Seirocrinus_subangularis* | Jurassic | Toarcian | Pentacrinoidea | Articulata | 1 | 2.05 | 0.23 | 0.11 | 13 |
| *Cupulocrinus_crossmani* | Ordovician | Katian | Pentacrinoidea | Cyathoformes | 1 | 3.88 | 0.33 | 0.09 | 12 |
| *Onychocrinus_ramulosus* | Carboniferous | Mississippian | Pentacrinoidea | Flexibilia | 0 | 3.00 | 0.00 | 0.00 | 11 |
| *Onychocrinus_ramulosus* | Carboniferous | Serpukhovian | Pentacrinoidea | Flexibilia | 1 | 3.21 | 0.42 | 0.13 | 11 |
| *Uintacrinus_socialis* | Cretaceous | Cretaceous | Pentacrinoidea | Articulata | 0 | 2.00 | 0.00 | 0.00 | 11 |
| *Cupulocrinus_plattevillensis* | Ordovician | Katian | Pentacrinoidea | Cyathoformes | 2 | 4.63 | 0.81 | 0.17 | 10 |
| *Onychocrinus_exsculptus* | Carboniferous | Serpukhovian | Pentacrinoidea | Flexibilia | 2 | 3.96 | 0.34 | 0.09 | 10 |
| *Chelocrinus_carnalli* | Triassic | Anisian | Pentacrinoidea | Articulata | 0 | 2.00 | 0.00 | 0.00 | 10 |
| *Chelocrinus_schlotheimi* | Triassic | Middle Triassic | Pentacrinoidea | Articulata | 0 | 2.00 | 0.00 | 0.00 | 10 |
| *Stuartwellercrinus_corbatoi* | Permian | Cisuralian | Pentacrinoidea | Cyathoformes | 0 | 1.00 | 0.00 | 0.00 | 9 |
| *Metacrinus_fossilis* | Paleogene | Eocene | Pentacrinoidea | Articulata | 2 | 4.69 | 0.52 | 0.11 | 9 |
| *Gymnocrinus_richeri* | Recent | Recent | Pentacrinoidea | Articulata | 0 | 1.00 | 0.00 | 0.00 | 9 |
| *Eoparisocrinus_crossmani* | Ordovician | Middle Ordovician | Pentacrinoidea | Cyathoformes | 1 | 3.21 | 0.43 | 0.13 | 8 |
| *Asaphocrinus_ornatus* | Silurian | Silurian | Pentacrinoidea | Flexibilia | 0 | 2.00 | 0.00 | 0.00 | 8 |
| *Pentacrinites_briareus* | Jurassic | Toarcian | Pentacrinoidea | Articulata | 0 | 2.00 | 0.00 | 0.00 | 8 |
| *Antedon_mediterraneous* | Recent | Recent | Pentacrinoidea | Articulata | 0 | 2.00 | 0.00 | 0.00 | 8 |
| *Comactina_echinoptera* | Recent | Recent | Pentacrinoidea | Articulata | 0 | 2.00 | 0.00 | 0.00 | 8 |
| *Saracrinus_angulatus* | Recent | Recent | Pentacrinoidea | Articulata | 2 | 4.08 | 0.36 | 0.09 | 8 |
| *Cupulocrinus_humilis* | Ordovician | Ordovician | Pentacrinoidea | Cyathoformes | 2 | 4.77 | 0.73 | 0.15 | 7 |
| *Cupulocrinus_humilis* | Ordovician | Rocklandian | Pentacrinoidea | Cyathoformes | 1 | 4.50 | 0.55 | 0.12 | 7 |
| *Dendrocrinus_casei* | Ordovician | Ordovician | Pentacrinoidea | Cyathoformes | 4 | 4.62 | 0.96 | 0.21 | 7 |
| *Botryocrinus_decadactylus* | Silurian | Silurian | Pentacrinoidea | Cyathoformes | 2 | 3.67 | 0.78 | 0.21 | 7 |
| *Eucalyptocrinites_crassus* | Silurian | Silurian | Camerata | Monobathrida | 1 | 2.03 | 0.17 | 0.08 | 7 |
| *Arthroacanta_carpenteri* | Devonian | Devonian | Camerata | Monobathrida | 0 | 2.00 | 0.00 | 0.00 | 7 |
| *Abrotocrinus_coreyi* | Carboniferous | Mississippian | Pentacrinoidea | Cyathoformes | 0 | 1.00 | 0.00 | 0.00 | 7 |
| *Dichocrinus_cinctus* | Carboniferous | Mississippian | Camerata | Monobathrida | 0 | 2.00 | 0.00 | 0.00 | 7 |
| *Dichocrinus_polydactylus* | Carboniferous | Serpukhovian | Camerata | Monobathrida | 0 | 2.00 | 0.00 | 0.00 | 7 |
| *Taxocrinus_colletti* | Carboniferous | Mississippian | Pentacrinoidea | Flexibilia | 0 | 3.00 | 0.00 | 0.00 | 7 |
| *Taxocrinus_colletti* | Carboniferous | Serpukhovian | Pentacrinoidea | Flexibilia | 0 | 3.00 | 0.00 | 0.00 | 7 |
| *Taxocrinus_meeki* | Carboniferous | Serpukhovian | Pentacrinoidea | Flexibilia | 0 | 3.00 | 0.00 | 0.00 | 7 |
| *Platycrinus_wachsmuthi* | Permian | Guadalupian | Camerata | Monobathrida | 0 | 1.00 | 0.00 | 0.00 | 7 |
| *Comathus_whlbergi_tenuibranchiu* | Recent | Recent | Pentacrinoidea | Articulata | 0 | 2.00 | 0.00 | 0.00 | 7 |
| *Diplocrinus_maclearanus* | Recent | Recent | Pentacrinoidea | Articulata | 0 | 2.00 | 0.00 | 0.00 | 7 |
| *Florometra_serratissima* | Recent | Recent | Pentacrinoidea | Articulata | 0 | 2.00 | 0.00 | 0.00 | 7 |
| *Isometra_graminea* | Recent | Recent | Pentacrinoidea | Articulata | 0 | 2.00 | 0.00 | 0.00 | 7 |
| *Psathyometra_fragilis* | Recent | Recent | Pentacrinoidea | Articulata | 0 | 2.00 | 0.00 | 0.00 | 7 |
| *Ectenocrinus_grandis* | Ordovician | Ordovician | Pentacrinoidea | Disparida | 0 | 2.00 | 0.00 | 0.00 | 6 |
| *Lecanocrinus_excavatus* | Silurian | Silurian | Pentacrinoidea | Flexibilia | 0 | 2.00 | 0.00 | 0.00 | 6 |
| *Hollowaycrinus_calvus* | Devonian | Lochkovian | Camerata | Diplobathrida | 1 | 2.08 | 0.29 | 0.14 | 6 |
| *Abrotocrinus_cymosus* | Carboniferous | Serpukhovian | Pentacrinoidea | Cyathoformes | 1 | 1.33 | 0.49 | 0.37 | 6 |
| *Barycrinus_rhombiferus* | Carboniferous | Mississippian | Pentacrinoidea | Cyathoformes | 4 | 2.33 | 1.13 | 0.48 | 6 |
| *Clathrocrinus_clathratus* | Carboniferous | Pennsylvanian | Pentacrinoidea | Cyathoformes | 0 | 1.00 | 0.00 | 0.00 | 6 |
| *Cyathocrinites_multibrachiatus* | Carboniferous | Mississippian | Pentacrinoidea | Cyathoformes | 5 | 3.67 | 1.56 | 0.42 | 6 |
| *Dichocrinus_inornatus* | Carboniferous | Mississippian | Camerata | Monobathrida | 0 | 2.00 | 0.00 | 0.00 | 6 |
| *Eratocrinus_salemensis* | Carboniferous | Mississippian | Pentacrinoidea | Cyathoformes | 3 | 1.33 | 0.84 | 0.63 | 6 |
| *Histocrinus_coreyi* | Carboniferous | Mississippian | Pentacrinoidea | Cyathoformes | 0 | 2.00 | 0.00 | 0.00 | 6 |
| *Sarocrinus_varsoviensis* | Carboniferous | Mississippian | Pentacrinoidea | Cyathoformes | 6 | 2.92 | 1.86 | 0.64 | 6 |
| *Zeacrinus_wortheni* | Carboniferous | Mississippian | Pentacrinoidea | Cyathoformes | 1 | 1.13 | 0.35 | 0.31 | 6 |
| *Erisocrinus_longwelli* | Permian | Cisuralian | Pentacrinoidea | Cyathoformes | 0 | 1.00 | 0.00 | 0.00 | 6 |
| *Erisocrinus_longwelli* | Permian | Permian | Pentacrinoidea | Cyathoformes | 0 | 1.00 | 0.00 | 0.00 | 6 |
| *Kiimetra_miocenica* | Neogene | Miocene | Pentacrinoidea | Articulata | 0 | 2.00 | 0.00 | 0.00 | 6 |
| *Amphimetra_ensifer* | Recent | Recent | Pentacrinoidea | Articulata | 0 | 2.00 | 0.00 | 0.00 | 6 |
| *Analcidometra_armatra* | Recent | Recent | Pentacrinoidea | Articulata | 0 | 2.00 | 0.00 | 0.00 | 6 |
| *Annacrinus_ wyvillethomsoni* | Recent | Recent | Pentacrinoidea | Articulata | 0 | 2.00 | 0.00 | 0.00 | 6 |
| *Bathycrinus_australis* | Recent | Recent | Pentacrinoidea | Articulata | 0 | 2.00 | 0.00 | 0.00 | 6 |
| *Endoxocrinus_parrae* | Recent | Recent | Pentacrinoidea | Articulata | 0 | 2.00 | 0.00 | 0.00 | 6 |
| *Metacrinus_levii* | Recent | Recent | Pentacrinoidea | Articulata | 2 | 6.74 | 0.59 | 0.09 | 6 |
| *Neocomatela_alata* | Recent | Recent | Pentacrinoidea | Articulata | 0 | 2.00 | 0.00 | 0.00 | 6 |
| *Tropometra_carinata_carinata* | Recent | Recent | Pentacrinoidea | Articulata | 0 | 2.00 | 0.00 | 0.00 | 6 |
| *Anomalocrinus_incurvus* | Ordovician | Ordovician | Pentacrinoidea | Disparida | 2 | 2.94 | 0.80 | 0.27 | 5 |
| *Lecanocrinus_macropetalus* | Silurian | Silurian | Pentacrinoidea | Flexibilia | 1 | 2.07 | 0.27 | 0.13 | 5 |
| *Codiacrinus_secundus* | Devonian | Lochkovian | Pentacrinoidea | Cyathoformes | 1 | 3.71 | 0.49 | 0.13 | 5 |
| *Dolatocrinus_lineolatus* | Devonian | Devonian | Camerata | Monobathrida | 0 | 2.00 | 0.00 | 0.00 | 5 |
| *Eutaxocrinus_whiteavesi* | Devonian | Devonian | Pentacrinoidea | Flexibilia | 1 | 2.78 | 0.43 | 0.15 | 5 |
| *Barycrinus_asteriscus* | Carboniferous | Mississippian | Pentacrinoidea | Cyathoformes | 0 | 2.00 | 0.00 | 0.00 | 5 |
| *Barycrinus_hoveyi* | Carboniferous | Mississippian | Pentacrinoidea | Cyathoformes | 0 | 2.00 | 0.00 | 0.00 | 5 |
| *Erisocrinus_typus* | Carboniferous | Pennsylvanian | Pentacrinoidea | Cyathoformes | 0 | 1.00 | 0.00 | 0.00 | 5 |
| *Moscovicrinus_multiplex* | Carboniferous | Carboniferous | Pentacrinoidea | Cyathoformes | 1 | 2.04 | 0.21 | 0.10 | 5 |
| *Onychocrinus_ulrichi* | Carboniferous | Carboniferous | Pentacrinoidea | Flexibilia | 0 | 4.00 | 0.00 | 0.00 | 5 |
| *Onychocrinus_ulrichi* | Carboniferous | Mississippian | Pentacrinoidea | Flexibilia | 2 | 4.00 | 0.41 | 0.10 | 5 |
| *Platycrinites_bozemanensis* | Carboniferous | Mississippian | Camerata | Monobathrida | 0 | 1.00 | 0.00 | 0.00 | 5 |
| *Platycrinites_hemisphaericus* | Carboniferous | Mississippian | Camerata | Monobathrida | 0 | 1.00 | 0.00 | 0.00 | 5 |
| *Platycrinites_hemisphaericus* | Carboniferous | Serpukhovian | Camerata | Monobathrida | 0 | 1.00 | 0.00 | 0.00 | 5 |
| *Arroyocrinus_popenoei* | Permian | Permian | Pentacrinoidea | Cyathoformes | 0 | 1.00 | 0.00 | 0.00 | 5 |
| *Metacrinus_serratus* | Recent | Recent | Pentacrinoidea | Articulata | 3 | 6.76 | 0.75 | 0.11 | 5 |
| *Neocrinus_blakei* | Recent | Recent | Pentacrinoidea | Articulata | 0 | 2.00 | 0.00 | 0.00 | 5 |
| *Praecupulocrinus_conjugans* | Ordovician | Middle Ordovician | Pentacrinoidea | Cyathoformes | 2 | 4.00 | 0.76 | 0.19 | 4 |
| *Pycnocrinus_altilis* | Ordovician | Late Ordovician | Camerata | Monobathrida | 0 | 2.00 | 0.00 | 0.00 | 4 |
| *Abacocrinus_sp* | Silurian | Wenlock | Camerata | Monobathrida | 0 | 2.00 | 0.00 | 0.00 | 4 |
| *Cyathocrinus_ramosus* | Silurian | Wenlock | Pentacrinoidea | Cyathoformes | 1 | 2.14 | 0.38 | 0.18 | 4 |
| *Periechocrinus_moniliformis* | Silurian | Wenlock | Camerata | Monobathrida | 0 | 2.00 | 0.00 | 0.00 | 4 |
| *Thylacocrinus_vannioti* | Devonian | Early Devonian | Camerata | Diplobathrida | 1 | 1.83 | 0.41 | 0.22 | 4 |
| *Aerocrinus_inmaturus* | Carboniferous | Mississippian | Camerata | Monobathrida | 0 | 2.00 | 0.00 | 0.00 | 4 |
| *Clathrocrinus_clinatus* | Carboniferous | Pennsylvanian | Pentacrinoidea | Cyathoformes | 0 | 1.00 | 0.00 | 0.00 | 4 |
| *Scytalocrinus_grandis* | Carboniferous | Mississippian | Pentacrinoidea | Cyathoformes | 1 | 1.67 | 0.52 | 0.31 | 4 |
| *Stellarocrinus_sp* | Carboniferous | Pennsylvanian | Pentacrinoidea | Cyathoformes | 0 | 1.00 | 0.00 | 0.00 | 4 |
| *Taxocrinus_wortheni* | Carboniferous | Serpukhovian | Pentacrinoidea | Flexibilia | 0 | 3.00 | 0.00 | 0.00 | 4 |
| *Neoplatrycrinus_dilatatus* | Permian | Guadalupian | Camerata | Monobathrida | 0 | 1.00 | 0.00 | 0.00 | 4 |
| *Traumatocrinus_sp* | Triassic | Late Triassic | Pentacrinoidea | Articulata | 1 | 1.57 | 0.53 | 0.34 | 4 |
| *Metacrinus_interruptus* | Recent | Recent | Pentacrinoidea | Articulata | 3 | 6.64 | 0.92 | 0.14 | 4 |
| *Archaetaxocrinus_burfordi* | Ordovician | Middle Ordovician | Pentacrinoidea | Flexibilia | 0 | 3.00 | 0.00 | 0.00 | 3 |
| *Cupulocrinus_gracilis* | Ordovician | Middle Ordovician | Pentacrinoidea | Cyathoformes | 0 | 4.00 | 0.00 | 0.00 | 3 |
| *Ectenocrinus_simplex* | Ordovician | Middle Ordovician | Pentacrinoidea | Disparida | 0 | 3.00 | 0.00 | 0.00 | 3 |
| *Nassoviocrinus_longibrachiatus* | Silurian | Ludlow | Pentacrinoidea | Cyathoformes | 1 | 4.33 | 0.58 | 0.13 | 3 |
| *Antihomocrinus_chapmani* | Devonian | Early Devonian | Pentacrinoidea | Cyathoformes | 1 | 3.33 | 0.58 | 0.17 | 3 |
| *Brabeocrinus_christinae* | Carboniferous | Pennsylvanian | Pentacrinoidea | Cyathoformes | 0 | 1.00 | 0.00 | 0.00 | 3 |
| *Decadocrinus_depressus* | Carboniferous | Mississippian | Pentacrinoidea | Cyathoformes | 0 | 1.00 | 0.00 | 0.00 | 3 |
| *Exocrinus_wanni* | Carboniferous | Pennsylvanian | Pentacrinoidea | Cyathoformes | 0 | 1.00 | 0.00 | 0.00 | 3 |
| *Hypselocrinus_indianaensis* | Carboniferous | Mississippian | Pentacrinoidea | Cyathoformes | 1 | 1.60 | 0.55 | 0.34 | 3 |
| *Oklahomacrinus_loeblichi* | Carboniferous | Missourian | Pentacrinoidea | Cyathoformes | 0 | 2.00 | 0.00 | 0.00 | 3 |
| *Syntomocrinus_sundaicus* | Permian | Guadalupian | Pentacrinoidea | Flexibilia | 0 | 2.00 | 0.00 | 0.00 | 3 |
| *Cassianocrinus_varians* | Triassic | Ladinian | Pentacrinoidea | Articulata | 1 | 1.78 | 0.44 | 0.25 | 3 |
| *Isocrinus_hanaii* | Cretaceous | Aptian | Pentacrinoidea | Disparida | 0 | 2.00 | 0.00 | 0.00 | 3 |
| *Isocrinus_nehalemensis* | Neogene | Oligocene | Pentacrinoidea | Disparida | 0 | 2.00 | 0.00 | 0.00 | 3 |
| *Metacrinu_musortomae* | Recent | Recent | Pentacrinoidea | Articulata | 3 | 5.27 | 1.01 | 0.19 | 3 |
| *Columbicrinus_crassus* | Ordovician | Middle Ordovician | Pentacrinoidea | Disparida | 0 | 4.00 | 0.00 | 0.00 | 2 |
| *Dendrocrinus_springeri* | Ordovician | Middle Ordovician | Pentacrinoidea | Cyathoformes | 0 | 6.00 | 0.00 | 0.00 | 2 |
| *Periechocrinus_sp* | Silurian | Wenlock | Camerata | Monobathrida | 0 | 2.00 | 0.00 | 0.00 | 2 |
| *Sagenocrinites_sp* | Silurian | Wenlock | Pentacrinoidea | Flexibilia | 1 | 2.25 | 0.50 | 0.22 | 2 |
| *Ophiocrinus_stangeri* | Devonian | Early Devonian | Camerata | Diplobathrida | 0 | 2.00 | 0.00 | 0.00 | 2 |
| *Barycrinus_lyoni* | Carboniferous | Carboniferous | Pentacrinoidea | Cyathoformes | 0 | 3.00 | 0.00 | 0.00 | 2 |
| *Cyathocrinites_sp* | Carboniferous | Serpukhovian | Pentacrinoidea | Cyathoformes | 2 | 2.40 | 0.89 | 0.37 | 2 |
| *Cyathocrinus_arboreus* | Carboniferous | Mississippian | Pentacrinoidea | Cyathoformes | 1 | 3.50 | 0.71 | 0.20 | 2 |
| *Euonychocrinus_simplex* | Carboniferous | Pennsylvanian | Pentacrinoidea | Flexibilia | 0 | 2.00 | 0.00 | 0.00 | 2 |
| *Histocrinus_graphicus* | Carboniferous | Mississippian | Pentacrinoidea | Cyathoformes | 0 | 2.00 | 0.00 | 0.00 | 2 |
| *Laudonocrinus_subsinuatus* | Carboniferous | Pennsylvanian | Pentacrinoidea | Cyathoformes | 0 | 1.00 | 0.00 | 0.00 | 2 |
| *Macrocrinus_mundulus* | Carboniferous | Mississippian | Camerata | Monobathrida | 0 | 2.00 | 0.00 | 0.00 | 2 |
| *Microcaracrinus_conjugulus* | Carboniferous | Pennsylvanian | Pentacrinoidea | Cyathoformes | 0 | 1.00 | 0.00 | 0.00 | 2 |
| *Onychocrinus_monroensis* | Carboniferous | Mississippian | Pentacrinoidea | Flexibilia | 1 | 3.50 | 0.58 | 0.16 | 2 |
| *Pachylocrinus_gibsoni* | Carboniferous | Mississippian | Pentacrinoidea | Cyathoformes | 0 | 2.00 | 0.00 | 0.00 | 2 |
| *Pachylocrinus_subaequalis* | Carboniferous | Tournaisian | Pentacrinoidea | Cyathoformes | 0 | 2.00 | 0.00 | 0.00 | 2 |
| *Paramphicrinus_oklahomaensis* | Carboniferous | Pennsylvanian | Pentacrinoidea | Flexibilia | 0 | 2.00 | 0.00 | 0.00 | 2 |
| *Pellecrinus_hexadactylus* | Carboniferous | Mississippian | Pentacrinoidea | Cyathoformes | 0 | 3.00 | 0.00 | 0.00 | 2 |
| *Plummericrinus_striatus* | Carboniferous | Missourian | Pentacrinoidea | Cyathoformes | 0 | 1.00 | 0.00 | 0.00 | 2 |
| *Scytalocrinus_disparilis* | Carboniferous | Mississippian | Pentacrinoidea | Cyathoformes | 0 | 1.00 | 0.00 | 0.00 | 2 |
| *Stellarocrinus_virgilensis* | Carboniferous | Pennsylvanian | Pentacrinoidea | Cyathoformes | 0 | 1.00 | 0.00 | 0.00 | 2 |
| *Taxocrinus_nobilis* | Carboniferous | Carboniferous | Pentacrinoidea | Flexibilia | 1 | 2.50 | 0.58 | 0.23 | 2 |
| *Graphiocrinus_timoricus* | Permian | Guadalupian | Pentacrinoidea | Cyathoformes | 0 | 1.00 | 0.00 | 0.00 | 2 |
| *Moapacrinus_inornatus* | Permian | Cisuralian | Pentacrinoidea | Cyathoformes | 0 | 1.00 | 0.00 | 0.00 | 2 |
| *Neoplatrycrinus_major* | Permian | Guadalupian | Camerata | Monobathrida | 0 | 1.00 | 0.00 | 0.00 | 2 |
| *Timorocrinus_mirabilis* | Permian | Guadalupian | Pentacrinoidea | Cyathoformes | 0 | 1.00 | 0.00 | 0.00 | 2 |
| *Apiocrinus_roissyanus* | Jurassic | Oxfordian | Pentacrinoidea | Articulata | 0 | 1.00 | 0.00 | 0.00 | 2 |
| *Balanocrinus_gracilis* | Jurassic | Early Jurassic | Pentacrinoidea | Articulata | 0 | 2.00 | 0.00 | 0.00 | 2 |
| *Pentacrinites_sp* | Jurassic | Early Jurassic | Pentacrinoidea | Articulata | 0 | 2.00 | 0.00 | 0.00 | 2 |
| *Pentacrinus_briaroides* | Jurassic | Early Jurassic | Pentacrinoidea | Articulata | 0 | 2.00 | 0.00 | 0.00 | 2 |
| *Pentacrinus_hiemeri* | Jurassic | Early Jurassic | Pentacrinoidea | Articulata | 0 | 2.00 | 0.00 | 0.00 | 2 |
| *Notocrinus_rasmusseni* | Paleogene | Eocene | Pentacrinoidea |  | 1 | 2.33 | 0.58 | 0.25 | 2 |
| *Isocrinus_oregonensis* | Neogene | Oligocene | Pentacrinoidea | Disparida | 1 | 1.67 | 0.58 | 0.35 | 2 |
| *Archaetaxocrinus_lanei* | Ordovician | Middle Ordovician | Pentacrinoidea | Flexibilia | 0 | 3.00 | 0.00 | 0.00 | 1 |
| *Delgadocrinus_oportovinum* | Ordovician | Middle Ordovician | Camerata | Monobathrida | 0 | 2.00 | 0.00 | 0.00 | 1 |
| *Camarocrinus_sp* | Silurian | Pridoli | Camerata | Monobathrida | 0 | 2.00 | NA | NA | 1 |
| *Carpocrinus_simplex* | Silurian | Silurian | Camerata | Monobathrida | 0 | 2.00 | 0.00 | 0.00 | 1 |
| *Cyathocrinites_acinotubus* | Silurian | Wenlock | Pentacrinoidea | Cyathoformes | 0 | 3.00 | 0.00 | 0.00 | 1 |
| *Cyathocrinites_capillaris* | Silurian | Wenlock | Pentacrinoidea | Cyathoformes | 0 | 2.00 | 0.00 | 0.00 | 1 |
| *Cyathocrinites_goniodactylus* | Silurian | Wenlock | Pentacrinoidea | Cyathoformes | 1 | 1.67 | 0.58 | 0.35 | 1 |
| *Dendrocrinus_arrugius* | Silurian | Ludlow | Pentacrinoidea | Cyathoformes | 0 | 6.00 | NA | NA | 1 |
| *Desmidocrinus_heterodactylus* | Silurian | Wenlock | Camerata | Monobathrida | 0 | 2.00 | 0.00 | 0.00 | 1 |
| *Gissocrinus_goniodactylus* | Silurian | Wenlock | Pentacrinoidea | Cyathoformes | 0 | 1.00 | 0.00 | 0.00 | 1 |
| *Nexocrinus_wallanensis* | Silurian | Pridoli | Camerata | Diplobathrida | 0 | 2.00 | NA | NA | 1 |
| *Arthroacanta_carpenteri* | Devonian | Middle Devonian | Camerata | Monobathrida | 0 | 2.00 | NA | NA | 1 |
| *Ophiocrinus_nnettae* | Devonian | Lochkovian | Camerata | Diplobathrida | 0 | 2.00 | 0.00 | 0.00 | 1 |
| *Sacrinus_hexensis* | Devonian | Early Devonian | Pentacrinoidea | Cyathoformes | 0 | 6.00 | NA | NA | 1 |
| *Stewbrecrinus_terryi* | Devonian | Lochkovian | Pentacrinoidea | Cyathoformes | 0 | 4.00 | NA | NA | 1 |
| *Abatocrinus_grandis* | Carboniferous | Mississippian | Camerata | Monobathrida | 0 | 2.00 | 0.00 | 0.00 | 1 |
| *Abrotocrinus_springeri* | Carboniferous | Mississippian | Pentacrinoidea | Cyathoformes | 0 | 1.00 | NA | NA | 1 |
| *Agaricocrinus_splendens* | Carboniferous | Mississippian | Pentacrinoidea | Monobathrida | 0 | 1.00 | 0.00 | 0.00 | 1 |
| *Alcimocrinus_girtyi* | Carboniferous | Atokan | Pentacrinoidea | Cyathoformes | 0 | 2.00 | 0.00 | 0.00 | 1 |
| *Apographiocrinus_typicalis* | Carboniferous | Pennsylvanian | Pentacrinoidea | Cyathoformes | 0 | 1.00 | 0.00 | 0.00 | 1 |
| *Atrapocrinus_mutatus* | Carboniferous | Atokan | Pentacrinoidea | Cyathoformes | 0 | 1.00 | 0.00 | 0.00 | 1 |
| *Barycrinus_neglectus* | Carboniferous | Mississippian | Pentacrinoidea | Cyathoformes | 0 | 4.00 | 0.00 | 0.00 | 1 |
| *Barycrinus_princeps* | Carboniferous | Mississippian | Pentacrinoidea | Cyathoformes | 0 | 2.00 | 0.00 | 0.00 | 1 |
| *Synbathocrinus_swallovi* | Carboniferous | Mississippian | Pentacrinoidea | Disparida | 0 | 2.00 | 0.00 | 0.00 | 1 |
| *Ciathrocrinus_grileyi* | Carboniferous | Atokan | Pentacrinoidea | Cyathoformes | 0 | 1.00 | 0.00 | 0.00 | 1 |
| *Contocrinus_coupi* | Carboniferous | Pennsylvanian | Pentacrinoidea | Cyathoformes | 0 | 1.00 | 0.00 | 0.00 | 1 |
| *Cyathocrinites_macrocrinus* | Carboniferous | Mississippian | Pentacrinoidea | Cyathoformes | 0 | 2.00 | NA | NA | 1 |
| *Cyathocrinites_opimus* | Carboniferous | Mississippian | Pentacrinoidea | Cyathoformes | 0 | 2.00 | 0.00 | 0.00 | 1 |
| *Cyathocrinites_poterium* | Carboniferous | Mississippian | Pentacrinoidea | Cyathoformes | 0 | 2.00 | 0.00 | 0.00 | 1 |
| *Cyathocrinus_angulatus* | Carboniferous | Serpukhovian | Pentacrinoidea | Cyathoformes | 0 | 2.00 | 0.00 | 0.00 | 1 |
| *Cydrocrinus_concinnus* | Carboniferous | Mississippian | Pentacrinoidea | Cyathoformes | 0 | 2.00 | 0.00 | 0.00 | 1 |
| *Decadocrinus_tumidulus* | Carboniferous | Mississippian | Pentacrinoidea | Cyathoformes | 1 | 2.50 | 0.71 | 0.28 | 1 |
| *Endelocrinus_tumidus* | Carboniferous | Pennsylvanian | Pentacrinoidea | Cyathoformes | 0 | 1.00 | NA | NA | 1 |
| *Eretmocrinus_commendabilis* | Carboniferous | Mississippian | Camerata | Monobathrida | 0 | 2.00 | 0.00 | 0.00 | 1 |
| *Exocrinus_sp* | Carboniferous | Missourian | Pentacrinoidea | Cyathoformes | 0 | 1.00 | 0.00 | 0.00 | 1 |
| *Exoriocrinus_lasallensis* | Carboniferous | Pennsylvanian | Pentacrinoidea | Cyathoformes | 0 | 1.00 | 0.00 | 0.00 | 1 |
| *Exoriocrinus_rugosus* | Carboniferous | Missourian | Pentacrinoidea | Cyathoformes | 0 | 1.00 | NA | NA | 1 |
| *Glaukosocrinus_planus* | Carboniferous | Missourian | Pentacrinoidea | Cyathoformes | 0 | 1.00 | 0.00 | 0.00 | 1 |
| *Graphiocrinus_mcadamsi* | Carboniferous | Mississippian | Pentacrinoidea | Cyathoformes | 0 | 1.00 | 0.00 | 0.00 | 1 |
| *Graphiocrinus_sp* | Carboniferous | Mississippian | Pentacrinoidea | Cyathoformes | 0 | 1.00 | 0.00 | 0.00 | 1 |
| *Haeretocrinus_wagneri* | Carboniferous | Pennsylvanian | Pentacrinoidea | Cyathoformes | 0 | 1.00 | 0.00 | 0.00 | 1 |
| *Hypselocrinus_hoveyi* | Carboniferous | Mississippian | Pentacrinoidea | Cyathoformes | 0 | 1.00 | 0.00 | 0.00 | 1 |
| *Moundocrinus_osagensis* | Carboniferous | Pennsylvanian | Pentacrinoidea | Cyathoformes | 0 | 1.00 | NA | NA | 1 |
| *Oklahornacrinus_frostae* | Carboniferous | Atokan | Pentacrinoidea | Cyathoformes | 0 | 2.00 | 0.00 | 0.00 | 1 |
| *Onychocrinus_exculptus* | Carboniferous | Mississippian | Pentacrinoidea | Flexibilia | 0 | 4.00 | 0.00 | 0.00 | 1 |
| *Pachylocrinus_manus* | Carboniferous | Mississippian | Pentacrinoidea | Cyathoformes | 0 | 1.00 | 0.00 | 0.00 | 1 |
| *Paradichocrinus_polydactylus* | Carboniferous | Mississippian | Camerata | Monobathrida | 0 | 2.00 | 0.00 | 0.00 | 1 |
| *Parisocrinus_crawfordsvillensis* | Carboniferous | Mississippian | Pentacrinoidea | Cyathoformes | 0 | 3.00 | 0.00 | 0.00 | 1 |
| *Parulocrinus_pontiacensis* | Carboniferous | Pennsylvanian | Pentacrinoidea | Cyathoformes | 0 | 1.00 | 0.00 | 0.00 | 1 |
| *Platycrinites_shumardanus* | Carboniferous | Serpukhovian | Camerata | Monobathrida | 0 | 1.00 | NA | NA | 1 |
| *Plummericrinus_credos* | Carboniferous | Pennsylvanian | Pentacrinoidea | Cyathoformes | 0 | 1.00 | 0.00 | 0.00 | 1 |
| *Proallosocrinus_exemptus* | Carboniferous | Atokan | Pentacrinoidea | Cyathoformes | 0 | 2.00 | 0.00 | 0.00 | 1 |
| *Probletocrinus_curtus* | Carboniferous | Pennsylvanian | Pentacrinoidea | Cyathoformes | 0 | 1.00 | 0.00 | 0.00 | 1 |
| *Sarocrinus_granilineus* | Carboniferous | Mississippian | Pentacrinoidea | Cyathoformes | 0 | 2.00 | NA | NA | 1 |
| *Springericrinus_magniventrus* | Carboniferous | Mississippian | Pentacrinoidea | Cyathoformes | 0 | 2.00 | 0.00 | 0.00 | 1 |
| *Stellarocrinus_bilineatus* | Carboniferous | Pennsylvanian | Pentacrinoidea | Cyathoformes | 0 | 1.00 | 0.00 | 0.00 | 1 |
| *Stenopecrinus_sp* | Carboniferous | Pennsylvanian | Pentacrinoidea | Cyathoformes | 0 | 1.00 | 0.00 | 0.00 | 1 |
| *Synerocrinus_incurous* | Carboniferous | Pennsylvanian | Pentacrinoidea | Flexibilia | 0 | 2.00 | 0.00 | 0.00 | 1 |
| *Taxocrinus_whitfieldi* | Carboniferous | Mississippian | Pentacrinoidea | Flexibilia | 0 | 3.00 | 0.00 | 0.00 | 1 |
| *Terpnocrinus_ocoyaensis* | Carboniferous | Pennsylvanian | Pentacrinoidea | Cyathoformes | 0 | 1.00 | 0.00 | 0.00 | 1 |
| *Ulocrinus_convexus* | Carboniferous | Pennsylvanian | Pentacrinoidea | Cyathoformes | 0 | 1.00 | 0.00 | 0.00 | 1 |
| *Uperocrinus_marinus* | Carboniferous | Mississippian | Camerata | Monobathrida | 0 | 2.00 | 0.00 | 0.00 | 1 |
| *Agnostocrinus_typus* | Permian | Cisuralian | Pentacrinoidea | Cyathoformes | 0 | 1.00 | 0.00 | 0.00 | 1 |
| *Anechocrinus_nalbiaensis* | Permian | Permian | Pentacrinoidea | Cyathoformes | 0 | 1.00 | NA | NA | 1 |
| *Archaeoisocrinus_occiduaustralis* | Permian | Permian | Pentacrinoidea | Articulata | 1 | 1.67 | 0.58 | 0.35 | 1 |
| *Arroyocrimus_popenoei* | Permian | Cisuralian | Pentacrinoidea | Cyathoformes | 0 | 1.00 | NA | NA | 1 |
| *Bursacrinus_magnificus* | Permian | Guadalupian | Pentacrinoidea | Cyathoformes | 0 | 1.00 | 0.00 | 0.00 | 1 |
| *Bursacrinus_procerus* | Permian | Guadalupian | Pentacrinoidea | Cyathoformes | 0 | 1.00 | 0.00 | 0.00 | 1 |
| *Bursacrinus_pyramidatus* | Permian | Guadalupian | Pentacrinoidea | Cyathoformes | 0 | 1.00 | 0.00 | 0.00 | 1 |
| *Calycocrinus_curvatus* | Permian | Guadalupian | Pentacrinoidea | Flexibilia | 0 | 3.00 | 0.00 | 0.00 | 1 |
| *Calycocrinus_malaianus* | Permian | Guadalupian | Pentacrinoidea | Flexibilia | 0 | 2.00 | 0.00 | 0.00 | 1 |
| *Calycocrinus_spinosus* | Permian | Guadalupian | Pentacrinoidea | Flexibilia | 0 | 3.00 | 0.00 | 0.00 | 1 |
| *Delocrinus_malaianus* | Permian | Guadalupian | Pentacrinoidea | Cyathoformes | 0 | 1.00 | 0.00 | 0.00 | 1 |
| *Elibatocrinus_elongatus* | Permian | Cisuralian | Pentacrinoidea | Cyathoformes | 0 | 1.00 | 0.00 | 0.00 | 1 |
| *Exocrinus_moorei* | Permian | Cisuralian | Pentacrinoidea | Cyathoformes | 0 | 1.00 | 0.00 | 0.00 | 1 |
| *Loxocrinus_globulus* | Permian | Guadalupian | Pentacrinoidea | Flexibilia | 0 | 2.00 | 0.00 | 0.00 | 1 |
| *Meganotocrinus_princeps* | Permian | Permian | Pentacrinoidea | Incertae sedis | 0 | 2.00 | NA | NA | 1 |
| *Moapacrinus_rotundatus* | Permian | Cisuralian | Pentacrinoidea | Cyathoformes | 0 | 1.00 | 0.00 | 0.00 | 1 |
| *Notiocrinus_timoricus* | Permian | Permian | Pentacrinoidea | Cyathoformes | 0 | 1.00 | 0.00 | 0.00 | 1 |
| *Palaeoholopus_pretiosus* | Permian | Guadalupian | Pentacrinoidea | Flexibilia | 0 | 3.00 | NA | NA | 1 |
| *Perimestocrinus_nevadensis* | Permian | Cisuralian | Pentacrinoidea | Cyathoformes | 0 | 1.00 | NA | NA | 1 |
| *Perimestocrinus_oasis* | Permian | Cisuralian | Pentacrinoidea | Cyathoformes | 0 | 1.00 | 0.00 | 0.00 | 1 |
| *Pleurocrinus_depressus* | Permian | Guadalupian | Camerata | Monobathrida | 0 | 1.00 | 0.00 | 0.00 | 1 |
| *Pleurocrinus_goldfussi* | Permian | Guadalupian | Camerata | Monobathrida | 0 | 1.00 | 0.00 | 0.00 | 1 |
| *Pleurocrinus_spectabilis* | Permian | Guadalupian | Camerata | Monobathrida | 0 | 1.00 | 0.00 | 0.00 | 1 |
| *Prophyllocrinus_cuspidatus* | Permian | Guadalupian | Pentacrinoidea | Flexibilia | 0 | 2.00 | NA | NA | 1 |
| *Prophyllocrinus_dentatus* | Permian | Guadalupian | Pentacrinoidea | Flexibilia | 0 | 2.00 | 0.00 | 0.00 | 1 |
| *Skaiocrinus_granulosus* | Permian | Permian | Pentacrinoidea | Cyathoformes | 0 | 1.00 | NA | NA | 1 |
| *Stachyocrinus_zea* | Permian | Guadalupian | Pentacrinoidea | Cyathoformes | 0 | 1.00 | 0.00 | 0.00 | 1 |
| *Stellarocrinus_complus* | Permian | Cisuralian | Pentacrinoidea | Cyathoformes | 0 | 1.00 | 0.00 | 0.00 | 1 |
| *Stomiocirnus_ferruginus* | Permian | Permian | Camerata | Monobathrida | 0 | 2.00 | 0.00 | 0.00 | 1 |
| *Synyphocrinus_permicus* | Permian | Cisuralian | Pentacrinoidea | Cyathoformes | 0 | 1.00 | NA | NA | 1 |
| *Thalassocrinus_gracilis* | Permian | Guadalupian | Pentacrinoidea | Articulata | 0 | 1.00 | NA | NA | 1 |
| *Timorocrinus_spinosus* | Permian | Guadalupian | Pentacrinoidea | Cyathoformes | 0 | 1.00 | 0.00 | 0.00 | 1 |
| *Dadocrinus_kunischi* | Triassic | Middle Triassic | Pentacrinoidea | Articulata | 0 | 2.00 | NA | NA | 1 |
| *Poteriocrinus_sp* | Triassic | Mississippian | Pentacrinoidea | Cyathoformes | 1 | 3.50 | 0.71 | 0.20 | 1 |
| *Apiocrinus_magnificus* | Jurassic | Oxfordian | Pentacrinoidea | Articulata | 0 | 1.00 | 0.00 | 0.00 | 1 |
| *Isocrinus_robustus* | Jurassic | Early Jurassic | Pentacrinoidea | Disparida | 0 | 2.00 | 0.00 | 0.00 | 1 |
| *Metacrinus_japonicus* | Recent | Recent | Pentacrinoidea | Articulata | 1 | 7.33 | 0.58 | 0.08 | 1 |
